# Supplementary material for: Are carbon emissions trading and green financial instruments synergistic? -Comprehensive quantitative research based on content analysis
Source: PLoS One. 2024 Mar 7;19(3):e0298601. doi: 10.1371/journal.pone.0298601 (PMC10919601; doi:10.1371/journal.pone.0298601)
Supplement: S1 Data — (ZIP) [file pone.0298601.s001.zip › Data/地级市Z_F工作报告.zip!/║┌┴·╜¡/║╫╕┌╩╨/└┤╫╘╣1⁄2╓┌║┼í╛┬φ┐╦╩2╛▌═°í┐_2012.docx]

各位代表：

　　现在，我代表市政府向大会作工作报告，请予审议。请政协委员和其他列席的同志提出意见。

　　一、过去五年工作回顾

　　本届政府任期的五年，是鹤岗历史上极不平凡的时期。这五年，我们在应对百年不遇国际金融危机的同时，更经受了有史以来最严重的旱涝灾害、多起矿难和森林火灾，以及造成重大影响的“集资借贷”事件等一系列严峻考验。这五年，我们在基本没有国家重大基础设施投资并承受诸多冲击的情况下，不仅圆满完成了既定任务，而且完成了城市发展的战略布局，启动了大批打基础、利长远的工程项目，并在多方面实现了重大突破，开创了我市更好更快发展的新局面。特别是在困难和危机面前，鹤岗人民表现出了空前的团结意识、驾驭能力和自强精神，作出了无愧于历史的贡献！

　　过去的五年，是综合实力不断提升的五年。2011年与2006年相比，地区生产总值从125亿元提高到300亿元，增长1.4倍；全口径财政收入从16亿元提高到48.8亿元，增长2倍，其中一般预算收入从7亿元提高到22亿元，增长2.1倍；固定资产投资完成137亿元，增长2倍；社会消费品零售总额实现83亿元，增长1.3倍；城镇人均可支配收入从8400 元增加到13850元，超过全省平均水平；农民实际人均纯收入从3780元增加到11330元，增长2倍，首次突破万元大关。特别是2011年，财政一般预算收入增幅列全省第三位，四煤城第一位，为历史最好水平；财政收入占地区生产总值的比重和产业投资占固定资产投资总额的比重，分别列全省第一位。各项主要指标增幅均高于全省平均水平，步入全省优秀行列。

　　过去的五年，是产业项目和园区建设实效明显的五年。全市持续开展 “项目建设年”、“四比竞赛”等活动，共实施千万元以上产业项目284个，完成投资224亿元，已开工和竣工项目总量超过改革开放以来近30年的总和。煤电化产业完成布局。中海油华鹤、龙煤东化2个大化肥项目和4个百万吨煤焦化及4个深加工项目，全部开工或投产，2个120万吨甲醇项目完成审批，从而结束了我市没有煤化工项目的历史。此外，龙煤立达90万千瓦矸石发电一期投产，60万千瓦热电联产项目报批；120万吨鸟山主焦煤项目、东北三省最大的400万吨峻发洗煤项目等开工建设。食品加工产业快速发展。万源、人和、泰丰等6个30万吨稻谷加工项目建成投产，全市稻米加工能力达到650万吨，占全省的1/5，并拥有3个中国驰名商标，加工深度居全省之首；兴汇集团100万吨玉米加工项目建成；全国单体产能最大的宝泉岭5万吨酱业扩产改造完成，全市大豆加工能力达到100万吨；双汇宝泉岭200万头生猪屠宰加工项目投产，全市生猪屠宰加工能力超过230万头。此外，全市日处理鲜奶能力达到700吨，木糖年加工能力达到1万吨。新兴产业项目迅速崛起。强力推进石墨产业发展，石墨储量、年加工量和出口量分别占全国的1/3、1/5和1/3，新建香港南海油等4个石墨项目，实现深加工零的突破。矿用救生舱、玉米剥皮机、联合转载机等一批拥有专利技术的机械制造项目开工建设；三精千鹤制药、双兰星制药、禾友农药等项目投产；100万吨鑫塔水泥等10个新型建材项目建成，特别是中岩抗裂合材料被联合国确定为永续性环保项目，并获得1.2亿元首批资金支持；双叶、翔鹤家具等林木深加工项目开工建设；萝北、绥滨风电及沐阳太阳能利用等清洁能源项目开工建设或投产。产业园区建设强力推进。确定了“两城一带十大园区”发展战略，“鹤南工业新城”、“中国石墨产业城”完成总体规划及红旗乡、环山乡行政区划调整，完成基础设施投资近10亿元。“鹤岗省级工业示范基地”获批，煤化工园区列入“省市共建重点园区”和“省财源建设示范园区”，并获得1亿多元建设资金。萝北和绥滨园区享受省级开发区政策，宝泉岭被省农垦总局列为“食品工业骨干区”，东山园区成为农业部、国台办批准的“台湾农民创业园”，南山园区被国家振兴东北办批准为再就业聚集区。其它园区也初具规模，从而我市实现了产业园区从无到多的重大突破，并为未来发展创造了充足空间。省委、省政府对我市的产业项目特别是煤化工园区建设给予了充分肯定，我市在省委召开的全省产业项目建设会议上介绍了经验，省政府东部煤电化基地产业项目建设现场会在我市召开。实践证明，我们把主要精力放在抓经济、抓产业发展上是正确的！

　　过去的五年，是产业结构不断优化的五年。三次产业结构调整到26∶49∶25；三次产业增加值分别实现78亿元、146亿元、76亿元，为2006年的1.7倍、2.2倍和1.6倍。工业经济显著增强。规模以上工业企业（可比口径）由35户增加到90户；税收实现30亿元，是2006年1.6倍。非公经济实现税收由2.3亿元增加到18.5亿元，是2006年的8倍；非公经济增加值占全市GDP比重由26%提高到38%，超过1/3。2011年，全市生产原煤1800多万吨、洗煤1300多万吨。龙煤鹤岗分子公司税收由4.2亿元增加到12.3亿元，增长2倍；地方煤矿在完成关闭34个矿井任务的情况下，实现税收由0.54亿元增加到2.8亿元，增长4倍。建筑营业税由0.67亿元增加到2亿元，增长2倍。现代农业加快建设。农业生产连年丰收，我市粮食产量从12亿斤增至25亿斤，域内粮食总产从52亿斤增至84亿斤，分别是2006年的2倍和1.6倍。全市水稻种植面积由50万亩增至 150多万亩，接近“过半”目标；有机、绿色、无公害种植面积达到170万亩，实现“过半”目标。在全省率先启动“农业机械化整市推进工程”，农业生产综合机械化程度由88%增至95%。在全省率先创建5个国家级标准化畜牧养殖示范场及11个养殖基地。农业产业化龙头企业由46户增至53户，被评为全省“农业产业化先进市”。基础设施不断加强，筹资近10亿元，实施了122个水利工程，江萝、绥滨等4大灌区开工建设，可新增水田灌溉面积180万亩；小鹤立河等7座水库、7座塘坝工程基本完成，新增旱涝保收田近100万亩。总投资26亿元的关门嘴子大型水库已列入国家重点支持项目。黑龙江流域现代农业科技示范园、绥滨船箱养鱼基地建成，五味子、蓝莓等基地总面积达到2.5万亩。新农村建设稳步发展，完成了52个省级示范村建设任务，农民专业合作组织由19个增至230个，增长11倍。第三产业迅速发展。积极争取“界江游”列入全省“十大旅游开发区”龙头景区，旅游产业快速启动。五年投入近20亿元，建成了龙江三峡、金顶山石林、太平沟黄金古镇、月牙湖风情园等20大景区和黑龙江流域博物馆等47个景点。名山旅游名镇一期工程建成并具备接待能力，岭北矿山地质公园、三峡森林公园、鹤岗森林公园、鹤北联营母树林公园被确定为国家级公园，6个景区被命名为国家级景区。创办了两届“国际界江旅游节”和首届“美食节”，组织了“黑龙江流域文明论坛”、“鹤岗名优特产品评展”等一系列文化、经贸活动。我市“北国边疆文化旅游区”被列入国家旅游“十二五”规划，并被授予“最佳山水旅游胜地”和“最具潜力旅游发展城市”等称号。接待国内外游客由50万人次增至近200万人次，增长3倍；旅游总收入由0.6亿元增至20亿元，增长32倍。深入实施“商务惠农”、“家电下乡”、社区“双进”工程，有6个社区被评为省和国家级示范区。积极引进大型企业，上海红星·美凯龙、香港七彩灯饰、吉林温馨鸟、江海证券、哈尔滨银行、龙江银行等十几个企业落户营业。完成《鹤岗现代物流业发展规划》，5个大中型物流项目开工建设。对外合作不断扩大。到2011年底，对俄合作项目累计达到41个，土地种植达到2万公顷，生猪养殖存栏2500多头，赴俄锰矿开采、节能灯项目即将投产，房地产开发项目全部竣工。与88个国家开展了贸易往来，进出口总额完成1.15亿美元、利用外资完成4600万美元，分别是2006年的4.4倍和22倍。

　　过去的五年，是城乡面貌显著变化的五年。确立了具有里程碑意义的“南兴、北开、东治、西拓、中升”五大城市发展战略，掀起城市建设新高潮，大力创建“三优文明城市”，城乡面貌极大改善，受到市内外广泛赞誉。大手笔推动新城建设。投入3000多万元，高标准完成了鹤南工业新城、松鹤生态新区、城市风貌特色及4个小城镇建设规划等。鹤南新城“三桥一路”等基础设施建成，松鹤新区“一河两路”等6项工程开工。实施“畅通工程”。编制了《鹤岗市区交通规划》，建成市货运中心和14个县乡级客运站，更新公交车200多辆。开通了鹤岗至天津列车，结束了没有出省列车的历史。投入17亿元，建成绥嘉等6条高等级公路，群众期盼多年的鹤名一级公路、东部绕城公路开工建设。投入6亿多元，完成1660公里农村公路建设，实现了“村村通”目标；投入3亿多元，完成130余条城市道路和10座城市桥梁建设任务。特别是投入2亿多元，一次性完成六个出城口改造任务，把昔日“脏乱差”的南大门改造成亮丽的“迎宾大道”。全省最长的富绥松花江大桥提前一年通车,全国最长的黑龙江固冰浮桥近日通车。鹤伊高速、绥萝公路、鹤名铁路、鹤佳铁路、铁路新站、鹤岗机场、文化高架桥等已开始规划设计。实施“四供两治”工程。大力实施“净水工程”，投资1.5亿元，完成了城市供水系统集中改造和五号水库污染源治理工程；建设农村饮水工程230多处，解决了14万人的饮水安全问题，农村自来水普及率由45%提高到76%。

大力实施“暖屋工程”，投资1亿多元完成了供热改扩建工程，新增供热面积718万平方米，完成了577栋旧楼供热设施和18个小区集中供热改造，基本解决了“弃供”问题。东西部2个污水处理厂、医疗废物处理项目竣工运行，生活垃圾处理工程开工建设，结束了我市没有污水、垃圾处理工程的历史。实施“五化”工程。加大硬化力度，新建或扩建了人民、鹤翔、振兴等14个城市广场和公园，新建小广场、小绿地116个，完成裸土治理76万平方米。加大绿化力度，义务植树完成1590万株，人工造林及封山育林完成50余万亩，完成大树进城1.5万余株，启动了集中绿化矿区、沉陷区、裸露山体和村屯任务，森林覆盖率、建成区绿化覆盖率、绿地率、人均公园绿地面积分别达到47%、43%、42%和15平方米，比2006年分别提高8%、15%、10%和3平方米。加大净化力度，集中开展了“除冰雪、清垃圾”和“小区环境整治会战”等活动。投入820万元，新建了13个水冲式厕所、2000多个卫生箱。投入4800万元，购置新型环卫设备117台（套），特别是2011年投入力度仅次于哈尔滨，位居全省第二位，环卫机械化水平快速提升，实现了城市卫生全天候保洁。加大亮化力度，编制了《鹤岗市重点区域亮化工程规划》，五年累计投资达2135万元，对重点建筑进行了亮化升级，全市亮灯率达到98%以上。加大美化力度，完成了467万平方米节能美容工程，被建设部列为全省唯一的“既有建筑节能改造示范市”。实施滨水城市工程。三年完成投资4.5亿元，“五湖一河十三沟”升级改造工程全面开工，其中天水湖公园投入使用，达到国内一流城市公园水平；清源湖、鹤立湖和小鹤立河沿岸景观带等工程加快建设；城区13条河沟防洪标准由不足5年一遇达到了20年以上。我市滨水城市建设工作在全省交流经验。实施城市功能提升工程。近三年，筹资6亿多元，新建了20个“中心”，其中，黑龙江煤电化高层人才培训中心、国省级鹤岗检测检验中心、公共资源公开交易中心、城市应急指挥中心、老干部活动中心、全民健身中心等18个中心建成并达到省内一流水平；区域医疗中心、青少年科技活动中心今年竣工。这20个中心的建成，极大提升了城市功能和品位。

　　过去的五年，是向上争取成果突出的五年。在争取政策支持方面，继我市被列入全省东部煤电化基地、两大平原农业开发区和北国风光特色旅游开发区之后，经过艰苦努力，又被列入国家“大小兴安岭林区生态保护与经济转型规划”和“资源城市转型试点市”，将获得重大支持,首批3.2亿元资金已到位。在争取资金支持方面，共获得40多亿元国家扩大内需等资金支持。其中，龙煤东化获得1.6亿元，成为东北三省支持最多的产业项目；既有建筑节能改造工程争取资金2.8亿元，居全省首位；土地矿山治理工程争取资金2.5亿元，居全省第三位；廉租房争取资金5.8亿元，居四煤城首位。在争取项目支持方面，在国家政策调整前，我市10个“百万级”大项目抢先获批。此外，还有涉及社会事业、基础设施等400多个项目列入国家和省支持计划。

　　过去的五年，是社会事业加快发展的五年。各类教育综合发展。省级标准化学校由13所增至39所，5所学校成为省级样板学校。小学和初中巩固率、高中毛入学率、高考升学率等进一步提高；新建18所学校教学设施、3所校外活动中心。启动校舍安全工程，城市学校D级危房改造全部完成。加强学前教育，2011年当年完成新建改建8所公办幼儿园。鹤岗师专通过教育部评估，毕业生就业率、专升本考取率连续四年高于全省平均水平。农村义务教育经费全部纳入财政保障范围，全面落实了中等职业学校和农村困难家庭学生学费、生活费补助政策。五年投入资金2180万元资助4万多名困难学生，兑现了不让全市一个孩子因贫失学的承诺。卫生服务日趋完善。医药卫生体制改革全面实施，医改工作经验在全国推广。建成卫校实验楼、职业病防治院等7所医疗和教学设施，改造城乡医院30所、惠民医院7所、县乡医疗卫生机构18个、社区卫生服务中心18个，社区卫生服务机构覆盖率达到93%。实施了“婴幼儿健康促进计划”、“惠家工程”和65岁以上老年人体检等一系列免费政策。市人民医院、鹤矿总医院晋升为国家“三甲”医院。科技创新不断增强。共实施高新技术项目458项，争取国家、省级科技计划项目52项，形成科技成果204项，获得国家专利340项。省和国家级高新技术企业达到23户，省级创新型企业达到11户,新建科技创新平台和研发机构17个，有5个项目列入国家星火计划等，科技成果转化率达到71%。文体事业成果显著。共投入2.1亿元，新建市博物馆、地质博物馆、数字电影院等8个文化场所、20个社区图书室和150多个农家书屋，乡镇文化场所实现了全覆盖；有线电视覆盖率由50%达到95%。文艺创作获得120多个省级以上奖项。积极开展全民健身运动，建成国民体质监测站、国家级田径短跑后备人才基地、全民健身主题公园。成功举办了机关运动会、市九运会、首届全民健身运动会，承办了全省摔跤锦标赛、全国中学生篮球赛等活动。竞技体育水平不断提高，获省级以上赛事金牌331枚，连续两届在省运会上取得金牌总数和团体总分第五名的好成绩。环境保护取得实效。设立每年1000万元的专项资金，实施减排工程55项。完成了重点污染企业鑫塔水泥整体搬迁和华能电厂脱硫工程，关停了城中10户小造纸厂和城边 6户白灰厂，37户洗煤企业全部实施废水循环改造，治理矿山23处，新建3个省级自然保护区。全面完成了国家下达的减排指标。

　　过去的五年，是人民群众实惠最多的五年。启动十大民生工程，财力投入大幅增加，五年办成单项投资百万元以上实事达到508项。积极促进就业创业。全面实施“千人创业”计划、“百名大学生就业创业工程”和“妇女创业就业工程”，发放公益性岗位和灵活就业补贴3.7亿元。全市新增就业10.8万人，城镇登记失业率控制在4.3%以内，高校毕业生就业率达到85%以上，保持了“零就业家庭”动态为零。不断完善社会保障体系。努力提高各类人群保障水平，失业保险标准由196元提高到490元，企业退休人员养老金标准由1164元提高到1296元，城市低保年人均补差额由1044元提高到2796元，农村低保年人均补差额由450元提高到1050元，均列全省前三位。争取2.7亿元，将2.5万名关停破产企业人员和7300名困难企业退休人员全部纳入医保，将2700名企业老工伤人员全部纳入工伤保险。城市人均公共卫生服务经费由7元提高到25元，城乡居民医保补助水平由80元提高到200元，社区工作者生活补贴由400元提高到1000元。基本药物制度试点全面推行，实现“一卡就医、即时结算”。建成儿童福利院，孤儿和散居孤儿生活补助标准分别达到1000元和600元。新建老年公寓51所、养老机构131个。对1.7万户低保户发放物价调节基金750万元，居全省第四。完成了56个村整村脱贫任务，城乡低收入和贫困人口由13.6万人下降到4.5万人。全市在岗职工年平均工资由1.6万元提高到3.5万元。机关事业单位公积金、医保和供暖补贴等达到全省上游水平，国家政策允许的各项工资和津贴补贴全部兑现，仅2011年人均月增资额就超过了500元。社保水平在全省排列后三位的全部晋位，并有多项步入中上游水平。大力实施安居工程。总投资14亿元涉及1.6万户的沉陷区治理工程全部完成。启动了涉及6.7万户的煤矿棚户区改造工程，已开工住宅306万平方米，竣工150万平方米；涉及1.66万户的农村泥草房改造工程，已完成1.3万户；涉及1700多户的国有林场危旧房改造工程已完成1000户。一氧化碳渗出区居民搬迁已达到650户。建成廉租房、公租房4500多套，低收入家庭每平方米年租金仅为6元；在全国率先推行了独生子女特扶家庭廉租房保障制度。全市人均住房面积从22.3平方米增至25.4平方米，在全省位于上游水平。积极创建平安城市。坚持开展“安全生产年”等活动，地方煤矿连续三年未发生3人以上重大事故，被评为全省先进单位；电力安全运行位列全国第二；城市消防连续15年无重大火灾事故，连续6年保持无人亡纪录，被评为全国示范单位。食品药品安全形势明显好转，查处造假案件工作受到国家食品药品监督局嘉奖。高度重视社会稳定，信访形势逐步好转。坚持从实际出发，妥善处置“集资借贷”问题，到2011年底，累计还款总数、全部还清欠款的企业数、全部还清集资款的群众数等三项指标分别达到了总数的80%。此外，累计对1.7万人次发放大病治疗和子女就学救助金3200多万元，处置工作受到国务院处非办和省委、省政府充分肯定。切实加强社会治安综合治理，先后开展了打黑除恶、打击黑车和酒驾等160余次专项活动，建立了“军警民”大巡防机制；刑事案件发案同比下降18%，现行命案破案率连续两年达到100%。新建或改建了10个基层派出所，建成了“110”指挥中心、第二看守所、第四消防中队和全省技术领先的实验室，在全省率先完成了市县两级应急通讯系统建设。

　　过去的五年，是政府改革建设不断增强的五年。顺利完成了政府机构等项改革，实行了一系列政府管理创新重大举措，并出台了政府系统改革创新奖励制度。在规划政策制定上，编制了“十二五”规划等35个中长期发展规划；出台了《城市资源经营办法》等30个组合性政策措施，对全市促发展、保稳定起到了重要作用。在财政金融保障上，在全省首家完成政府平台公司制改革，为企业融资33亿元。特别是以民生为重点，投入4165万元设立了扶贫解困救助、特困学生救助、劳模解困救助等21项保障性基金，基金数量和保障范围居全省之首。全面加强金融工作，与7家金融机构实现战略合作；创办民营经济担保商会和10家小额贷款公司，累计为企业提供贷款11亿多元。全市金融机构存贷额，分别比2006年提高了1 倍以上，存贷比达到 65.3%，列全省第三位，四煤城第一位。在深化区域合作上，与省农垦总局、森工总局、龙煤集团、华能黑龙江公司和中海油化学公司签署了“4+1”区域合作协议；与北京、上海、湖北及东北东部十二市（州）等市区友好关系不断巩固；与省科技厅及省内外23所高校签订了合作协议。在推进效能建设上，出台了《市政府效能建设实施方案》等27个制度性文件，在政府系统全面推行“五治行动”，提倡“七种作风”，实施了目标管理和倒计时落实责任制等，撤消了108个政府议事机构，减少了86个审批事项，行政审批改革工作在全省、全国介绍经验。主动接受市人大、市政协和人民群众监督，共办理人大代表建议440件、政协委员提案1299件，办复率均达100％。高度重视科学决策，组建了“经济发展顾问组”和“城市建设专家组”；坚持问计于民、问需于民，连续两年通过媒体征求市民建议240多条。严格落实廉政责任制，对重要决策、重要人事、重点项目安排和大额资金使用等事项，取消“一支笔”审批，全部实行集体决策，公共资源交易、政府重点工作、领导职责分工等全部向社会公开，全省党务政务公开现场会议在我市召开。

　　此外，民族、宗教、外事、侨务、审计、监察、人防、司法、统计、地震、残联、老龄、档案等项事业都取得了新成绩。

　　各位代表，上述成绩是省委、省政府和市委正确领导、市人大和政协大力支持、几届班子不懈努力、区域各单元同心合力和全市人民共同奋斗的结果。在此，我代表市政府，向全市各族人民、各民主党派、工商联、各人民团体和各界人士，向中省直各单位、驻军官兵和所有支持帮助鹤岗发展的各级领导、各方友人表示衷心的感谢和崇高的敬意！

　　回顾这五年，我们也清醒地认识到，我市在经济社会发展中还存在诸多矛盾和问题：一是从经济发展看，经济总量偏小，产业结构层次较低等问题仍然突出；二是从社会建设看，城乡居民收入偏低，社会事业和城市基础设施欠账较多，民生有待进一步改善;三是从安全稳定看，群众上访问题依然严重，煤矿安全形势依然严峻;四是从政府工作看，一些领导干部和公职人员能力不强、作风不实、执行不力，对群众的问题解决不及时等问题仍然比较突出，等等。对此，我们必须高度重视，认真解决，切实提高政府的公信度和人民群众的满意度。

二、今后五年总体构想和2012年主要任务

　　今后五年，将是鹤岗改革开放攻坚期、经济发展跨跃期、民生水平提升期和城市转型机遇期，更是鹤岗乘势而上、大有作为的五年。

　　今后五年的预期目标是：到2016年，在保证“十二五”期末实现“712”目标的基础上，力争地区生产总值再增长10%以上，达到800亿元左右；财政收入再增长20%，达到120亿元左右；固定资产投资再增长30%，五年累计达到2500亿元左右。城乡居民收入比2011年翻一番，其中，城镇居民人均可支配收入由13850元提高到27700元左右，农民人均纯收入由11330元提高到 22600元左右。与此同时，城市七大定位、五大战略的重点工程基本完成，现代产业体系基本形成，城市面貌发生重大变化，人民生活水平显著提高，进而加速转型跨跃，奔向富裕和谐，开创鹤岗更好更快发展的全新局面。

　　2012年，是实现“十二五”规划的承前启后之年，也是新一届政府的开局起步之年，抓好今年的工作具有重大意义。

　　按照中央、省委经济工作会议和市第十一次党代会作出的战略部署，今年政府工作的总体要求是：坚持以科学发展观为统领，深入落实省委“八大经济区、十大工程”发展战略和我市“758”发展战略，坚持以经济建设为中心，以改革创新为动力，以城市转型为主线，以项目建设为支撑，以改善民生为根本，以安全稳定为保障，加快发展方式转变和经济结构调整，全力推进更好更快发展，为实现“十二五”规划和全面建设小康社会的目标奠定更加坚实的基础。

　　今年全市经济社会发展的底限指标是：地区生产总值增长13%，地方财政收入（按可比口径）增长18 %，固定资产投资增长30%，工业增加值增长20%，社会消费品零售总额增长17%,进出口总额和利用外资分别增长15%，城镇居民人均可支配收入和农民人均纯收入分别增长13%。同时，要按照“712”目标，力争实现更快增长，财政收入占地区生产总值的比重、税收收入占财政收入的比重、产业投资占固定资产投资的比重等体现发展质量和效益的指标稳步提升。

　　围绕上述目标，重点要在四个方面实现突破：

　　（一）以产业项目和园区建设为重点，在经济发展上实现新突破。深入开展“项目产业建设年”及“四比竞赛”活动，坚持靠项目促增长、优结构、转方式、增就业。今年，要以大批量上新项目、大批量建成投产为目标，重点实施100个千万元以上产业项目。加快推进煤电化工产业项目，要着力打造煤焦化、煤制化肥、煤制烯烃三条产业链。重点加快中海油华鹤化肥项目，争取明年投产。龙煤东化积极引进战略合作者，争取重新启动；万昌、宝源焦化项目完成主体工程，征楠、天富、汉兴等项目力争投产。鸟山煤矿完成基建主体工程，峻发洗煤等项目投产，城市热电联产项目和立达矸石发电二期力争开工。全力推进中石化180万吨甲醇转烯烃项目。加快推进优质绿色食品产业项目，兴汇粮食副产品循环加工、经纬木糖醇项目开工建设，万源、人和、三江、泰丰等7个粮食深加工项目和阿凌达、家富、信赢等3个生猪养殖加工项目年内投产。做大做优黑龙江、鹤立湖等特色水产养殖基地，建设鳇鱼、大白鱼等5个名特优养殖示范场。加快推进石墨深加工产业项目，积极引进战略投资者，中铁资源集团总投资78亿元的深加工项目力争开工建设。香港南海油等3个深加工项目投产，3个新项目开工建设。大力发展新兴产业项目，力争世达联合收割机、龙跃玉米剥皮机、长安支架、天鹤铝材等项目年内投产，华升汽车配件、龙士达重工机械、鹤丰救生舱等项目开工建设。重点推进广亿细木工板、富鑫实木门等3个项目投产达效，双叶、祥鹤等高档实木家具项目加快建设，万寿菊、五味子等北药开发项目和蓝莓、山野菜等林产品加工项目做成做大。同时，加快推进绥滨、萝北、鹤北风电和宝泉岭秸秆发电等项目建设。赴俄锰铁矿开采和节能灯具项目建成投产，境内电解锰和铁合金项目力争开工建设。启动300万吨钢联产项目。加快“两城一带十大园区”建设，要坚持一手抓基础设施，一手抓招商引资，努力形成项目集群、产业集聚、要素集约的发展格局。“鹤南工业新城”要打造成全市“大工业园区”，为各县区发展“飞地经济”、主城区实现“腾笼换鸟”和承接发达地区产业转移创造条件。重点抓好省市共建的煤化工园区，达到全省一流水平。“中国石墨产业城”要立足全市布局，完成产业发展总体规划，启动资源整装勘察，建成省级石墨产品检测中心和科技研发中心，争取成为省市共建重点园区和国家级研发中心，为产品深加工和配套产业发展创造条件。“东宝鹤萝绥产业带”要进一步完善总体规划，实施优惠政策，合理分配利益，充分调动各方积极性，“替代产业集聚区”形成规模。各级各类园区都要加快基础设施建设，不断提高承载力，重点完成工业新城、煤电化园区、高新技术产业园的路桥、供水、供电工程和园区综合服务中心建设。全市重点园区要基本实现“七通一平”，其它园区实现“五通一平”。为确保今年项目和园区建设取得实效，今年2月份将召开全市产业项目建设工作会议，3月底基本完成拟开工项目的各项准备工作，4月下旬首批项目集中开工剪彩，7月份第二批项目集中开工剪彩。举办第四届东北东部区域合作会议。

　　（二）以产业结构优化升级为重点，在城市转型上实现新突破。科学制定《鹤岗城市转型规划和发展战略》，坚持政府主导，组建城市转型办公室。坚持以产业结构调整为重点，以“一产做强、二产做精、三产做大、资源产业做深”为目标，着力打造“四轮驱动”的复合式转型模式，努力形成以经济转型为中心，以体制转型、社会转型、文化转型为保证的“四位一体”格局。

　　要坚持工业立市战略，大力发展新型工业。重点实施“抓项目提增量、抓企业提存量、抓结构提质量”三大举措，不断强化工业主导地位和企业主体地位。支持传统企业改造升级，鼓励企业提高自主创新和市场竞争能力。积极推进北方水泥扩能、宝泉酱业扩产、中稻技术改造、新北国啤酒搬迁改造和双兰星、禾友新品开发等项目，基本完成地方煤矿集团化整合。支持新建企业达产达效，组织开展全市项目建设“回头看”，围绕项目投产率、达产率、税收贡献率和就业贡献率开展考核评比活动，引导全市上下更加务实地抓好项目建设工作。支持骨干企业做大做强，进一步深化与鹤矿、宝局、鹤北局、华能鹤电和中海油等大企业合作，加快生产要素优化组合，提高区域综合竞争力。支持小微企业生存发展，全面贯彻国务院支持小微企业发展政策，落实小微企业税费减免措施。要着力帮助各类企业解决资金、资源、运输等突出问题，不断改善经济发展环境，建立中小企业服务中心及融资服务平台，开展第二批“星级企业”评选活动。全市现有规模以上企业利税增长达到15%以上，新增规模以上企业20户以上；非公企业增加值和税收分别提高16%以上，高新技术企业产值增加20%以上。

　　要不断巩固农业基础地位，大力发展现代农业。抢抓机遇，重点实施“六化两增”工程，大力在全省率先实现农业现代化和城乡一体化两大战略目标。大力推进水利现代化，抓紧完成全市《水利工程建设规划》，成立水利投融资公司；重点完成江萝、德龙灌区以及阿凌达除险加固等工程，做好关门嘴子水库、金顶山水库开工前准备工作；完成灌区和涝区水利工程，新增旱涝保收田15万亩。大力推进农业机械化，田间综合机械化水平提高到96%，达到全省先进水平。大力推进农业产业化，扶持龙头企业做大做强，创建4个农业集团公司，企业销售收入和利税均增长15%。大力推进种植业优质化，发展高效经济作物，提高粮食单产、总产和优质化率。水稻种植面积从150万亩达到165万亩左右，实现“过半”目标；有机、绿色、无公害食品种植面积从170万亩达到180万亩；杂粮、果蔬、饲草等优质高效作物达到7万亩，开工建设马铃薯繁育基地。大力推进畜牧水产养殖标准化，建设优质安全畜产品供给功能区和标准化现代养殖示范区，牧（渔）业产值和增加值增长8%以上。大力推进土地规模化，启动56个村整村土地经营规模化项目，实现规模经营120万亩。大力推进城乡一体化，启动以东山区为试点、以“九统一”为主要内容的城乡一体化工程。同时，积极引导农民发展劳务经济和多种经营,向二、三产业和城镇转移，在农业增产、农民增收方面实现新的跨跃。

　　要充分发挥新兴产业拉动作用，大力发展现代服务业。从实际出发，“坚持扩大内需、外需两个市场，突出现代、传统六个产业”。突出发展文化产业。编制《文化产业发展纲要》，加快文化体制机制改革创新，完成国有文艺院团改革，组建市文化演艺公司、鹤广文化传媒公司等。加快发展文化创意、文艺演出、休闲娱乐、广告传媒、节庆会展等十大文化产业，重点推进“龙江三峡”文化旅游集合区、太平沟黄金古镇和迦南星城影视基地项目建设，启动红色文化旅游集合区和文化创意产业基地建设。鼓励民营资本投资文化产业，重点扶持晟誉公司文化传媒、丽云公司石墨雕塑、奥兴公司煤雕、翰墨轩环保书画等企业，使其尽快形成产业规模，成为龙头企业。突出发展旅游产业。不断提升服务水平和经营能力，组建市旅游集团，启动旅游集散中心项目，建设30个标准化农家店，大力发展市民市郊旅游项目。推进名山旅游名镇二期工程，启动国家级滑雪场等项目；加快提升嘟噜河、绥滨两江湿地建设水平，做好湿地旅游文章。全方位扩大旅游产业知名度和竞争力，旅游人次和旅游收入分别增长20%和30%。突出发展现代物流和商贸产业。提升商业业态，大力改善生产和消费条件，争取时代广场、地下商业街、红星·美凯龙二期、宏益家电旗舰店和4个物流园区全部竣工，大商新玛特购物广场、新城购物中心开工建设，积极引进五星级酒店建设项目。突出发展金融业。实施金融强市战略，健全激励机制，打造诚信环境，推进农村信用社改革，稳步发展小额贷款公司，做好10亿元城市债券发行工作。金融机构储蓄和贷款余额分别增长 20%以上。突出发展对俄经贸。着力抓好大通道建设，启动第二港区建设规划，建成口岸商品集散地和购物中心。进一步扩大优质地产品出口，培育和提升企业进出口能力。实现口岸过客、过货分别增长20%、15%，争取进入全省前列。

　　（三）以改善民生为重点，在社会事业和城市建设上实现新突破。坚持以人民群众最关心、最直接、最现实、最急需的问题为重点，进一步加大财政投入力度，提高社会管理、公共服务水平，全面推进十大工程，切实抓好惠民利民的117件大事实事。

　　一是抓好文明城市建设工程。全面实施城市发展五大战略，争创省级“三优文明城”。加快推进“南兴”战略，对兴安区200平方公里版图进行统一规划，以60平方公里核心区为重点，以建设“鹤南工业新城”为目标，实行“特区”政策，实现全面放权，加快发展速度。大力改善兴安区区容区貌和基础设施，重点完成“鹤南滨水景观带”、迎宾大道、阿凌达湖升级改造工程和易灾区、污染区搬迁工程等，创造全省一流的“宜业”环境。加快推进“北开”战略，对东山、宝泉岭、鹤北、萝北、绥滨等北部区域产业发展和公共基础设施建设进行统一规划，以“开放大通道”、“接替产业带”建设为重点，全面加快对俄开放和农、林、水、游、矿、贸等六大产业开发。重点完成鹤名公路主体工程并力争竣工通车，加快推进鹤名铁路、鹤岗机场、绥萝公路前期工作。启动城市“北大门”环境综合整治工程，完成云山、绥东供电工程，加快推进农村电网升级改造，消灭无电村，全市实现国网供电。加快推进“东治”战略，对南山、东山、兴山等东部区域进行统一规划，以建设“绿色矿山城市”为目标，加快沉陷区、采空区、棚户区、一氧渗出区和污染企业治理，彻底改变东部恶劣的生产生活条件。重点完成石头河、西山沟改造升级工程，建成绕城公路一期，启动南山第三跨线桥等路桥规划建设。加快推进“西拓”战略，对东山、工农等100平方公里的西部区域进行统一规划，以25平方公里核心区为重点，以建设松鹤生态新区为目标，突出抓好小鹤立河扩容升级工程，使之成为鹤岗最秀美的穿城河流；加快建设湖滨大道等7项主路网工程，完成清源湖、鹤立湖和松鹤西湖基础设施项目，积极推进行政办公区、公职人员小区、鹤矿和华鹤职工生活区等项目建设，加快推进鹤佳铁路及新火车站、新客运站等项目前期工作，创造全省一流的“宜居”环境。加快推进“中升”战略，对向阳、工农、兴山、南山等中部区域进行统一规划，老城新区统筹推进，以主城区产业、功能、管理、服务、形象全面升级为重点，加快改善市民生活环境。全面提高“五化”水平，重点加快背街小巷和居民住宅区环境改造，完成10万平方米裸土治理任务，修建5条城市道路和90条街巷道，道路完好率达到95%以上。进一步提升绿化水平，科学种植乔灌花草，继续推进“大树进城”、“区片绿化”等工作，完成150万株义务植树任务。全面提高亮化标准，大力推广节能灯具，完成城市重点建筑物、路、桥及部分巷道亮化任务。进一步完善市内公园和广场基础设施，完成北山文化公园、岭南休闲广场等建设；继续实施既有建筑节能美容工程。全面提高城市管理和服务水平，推进市场化运作，鼓励引进大型物业公司，成立清雪公司等，重点抓好小区物业管理、垃圾冰雪清运、供热供气工程等。同时，进一步加快小城镇和新农村建设，完成全市重点小城镇建设规划，忠仁镇、新华镇、五道岗农场等重点工程全面开工，开展村容村貌整治评比竞赛活动。

　　二是抓好教育优先发展工程。以提高教育质量、促进教育公平为核心，优化整合教育资源，努力提高各级各类教育综合发展水平。加强学前教育，新建7所公办幼儿园；抓好义务教育，确保7所学校达到标准化学校，1所校外活动中心建成；发展职业教育，创建国家级重点中等职业学校和职业教育实训基地；提升师专办学水平，重点抓好特色专业建设；重视特殊教育，创建标准化特教学校。积极支持民办教育规范发展，办好一批高质量的民办学校和幼儿园。切实抓好校安工程，对煤城小学等7所中小学校进行重点规划建设，加大校车、学生就餐和校园危房监管整治力度，确保学生、学校安全。切实改善办学条件，全部完成学校供热管网改造和操场硬化任务，进一步解决任课教师、班主任编制和待遇问题。

　　三是抓好医疗卫生服务工程。以加快推进医药卫生体制改革为核心，以公立医院改革为重点，以健全四个保障体系为主线，全面提升公共卫生服务能力和突发事件应急处置能力。建成市医疗中心、急救中心和公共卫生突发事件应急指挥平台，启动市妇幼保健院、市精神病防治院迁建和食品药品、保健品、化妆品检验中心建设等工程，新建20个规范化村卫生所。深化人口和计划生育惠家工程，推进婴幼儿健康和生殖健康促进计划，兑现当年城镇无业人员独生子女父母奖励费，并积极解决历史拖欠问题。继续实施各项免费政策。

　　四是抓好文化体育繁荣工程。全面落实中央、省委部署，加快推进文化强市和文化惠民工程，努力满足人民群众精神文化生活。继续实施《三年文明建设规划》，大力弘扬“鹤岗精神”，加强道德建设，组织开展“道德模范”、“诚信企业”、“文化名人”等弘扬社会主义核心价值观的评选活动。深入开展乡镇综合文化站、街道文化中心、行政村和社区文化活动室达标创建工作；继续开展“欢乐鹤岗行”文化下基层等系列活动，启动首届“中俄界江之夏音乐会”筹备工作。加大文化设施建设力度，建成市博物馆、城市规划展馆，启动图书馆、群众艺术馆、妇女儿童活动中心、青年爱国主义教育基地等9个文化场馆建设工作。开播广播电视网站，购置电视转播直播车，升级改造电视播出系统，有线电视数字化率达到80%以上。将文化发展基金提高到100万元。深入落实《全民健身条例》，加大对公共体育设施投入，新建10个群众户外体育活动场所，启动松鹤新区体育场馆和冰雪运动设施建设，进一步提高竞技体育水平。

　　五是抓好人才科技强市工程。牢固树立“人才是第一资源”的理念，加大优秀人才培养和引进力度。落实“6465”人才培养计划、人才“双百”工程和社区专业化人才“百千万”工程，将人才教育基金提高到100万元。大力支持办好煤电化高层次人才培训中心暨黑科技研究生班、哈工大工商管理硕士鹤岗班和哈医大鹤岗研究生班，并开设4个专业实训基地。启动市委党校（行政学院）迁建工程、市技师学院筹建工作。充分发挥科技先导作用，提高优秀科研成果落地转化率。培育国家级高新技术企业2户、省级民营科技企业10户，新增企业研发机构5户，形成科技成果40项，申报国家专利10项以上。建成青少年科技活动中心，启动“数字鹤岗”地理信息建设工程。

　　六是抓好生态环境保护工程。强化节能减排目标责任制，更加注重资源与环境保护，严格执行项目环评制度。主城区和松鹤生态新区严禁有污染项目进入，已有污染企业逐步迁出。继续关闭严重污染企业，彻底拆除原鑫塔水泥厂，启动东华水泥厂搬迁、华能电厂脱硝工程，加快实施集中供热和烟尘治理工程，提高空气质量。完成岭北、峻德国家级绿色矿山申报工作。继续实施水土流失治理工程，新增治理面积2.4万亩。建成市区垃圾处理厂、向阳区垃圾填埋场和两县污水、垃圾处理工程。

　　七是抓好人民群众安居工程。以解决中低收入家庭住房为重点，努力实现全市人民“住有所居、居有改善”。煤矿棚户区改造开工建设住宅112万平方米，竣工100万平方米，安置居民1.5万户；鹤南新城、松鹤新区争创省级棚改示范小区。新建廉租住房2000套，安排100户计划生育特困家庭入住，为1.5万户以上居民发放租赁住房补贴；新建公共租赁住房200套。全部完成农村泥草房改造，实现“8年工程5年完成”的目标。完成水淹区及旧城区改造50万平方米，翻建3000户农村危房，完成“一氧渗出区”治理1000户。

　　八是抓好就业创业增收工程。坚持把就业创业摆上更加突出的位置，加快提高城乡居民收入水平，增加中等收入阶层比例，着力缩小贫富差距。全年实现新增和再就业2.3万人以上，城镇登记失业率控制在4.2%以内，保持“零就业家庭”动态为零。加强特殊群体就业救助，力争使90%以上的高校毕业生实现就业；实施“助残就业爱心工程”，开发残疾人公益岗位；为有创业愿望人员发放小额担保贷款5000万元。完成7个村整村脱贫，转移农村劳动力6万人次。继续实施创业带就业三年行动计划，全年培训1.3万人次。积极发展家庭服务业，对新办小企业实行税费减免政策。

　　九是抓好社会保障扩升工程。城乡居民最低生活保障、企业退休人员养老金、失业保险金待遇、企业最低工资、被征地农民新农保、农村“五保”供养等保障标准提高到全省先进水平。推行城镇居民社会养老保障，加快解决农林牧渔四场人员、刑满释放劳教人员参加养老保险问题。进一步深化殡葬改革，在全市免除基本殡葬费用。全面加强和谐社区建设，创建5个省级、20个市级充分就业社区。实施社区基础设施建设“双百工程”，新建或改扩建达标社区18个，城市社区达标率和农村社区建设启动率达到50%以上。

健全残疾人基层组织，推动残疾人社会保障和服务体系建设。切实加强物价监管，继续对低收入群体进行物价补贴，居民消费价格涨幅指数控制在4%左右。筹资3500万元，对困难群众开展“情暖万家”大型救助活动。继续落实“菜篮子”行政首长负责制，抓好蔬菜生产和仓储设施建设，建成500栋蔬菜温室大棚，蔬菜种植面积达到5.5万亩，努力让群众吃上低价菜和放心菜。

　　十是抓好平安城市创建工程。高度重视依法治市和城市安全稳定工作。进一步提升和加强创新社会管理工作水平，下大力气解决一批群众反映强烈的信访问题。坚持抓好“非法集资”后续处置工作，进一步加快还款进度，重点解决还款不平衡和恶意逃债问题；加大依法解决力度，对不能按要求还款的企业和个人，全部进入司法程序。深入开展“安全生产年”和“安全生产打非治违”专项治理等活动。以煤矿安全生产为重点，严格落实政府和企业两个主体责任，切实加强地矿“六大系统”和鹤矿国家级救援基地建设，严厉惩治各种违规违法行为。深入开展道路交通、消防森防和食品药品生产流通等重点领域专项整治，坚决遏制各类重大安全生产事故。不断完善城市监控网络体系建设，新增1000个监控设施，重点区域覆盖率达到97%；完成市区和两县42个派出所监控报警平台建设；实施公交、出租车辆交通安全GPS定位监控工程。加强各类灾害防御工作，建成地震应急避难场所12处，完成气象观测站迁址和灾害预警中心建设，开工建设人防指挥所，购置消防举高车等救援设备。坚决打击违法犯罪行为，保持命案必破，切实增强全市人民的安全感。

　　（四）以提升执政能力为重点，在政府自身建设上实现新突破。新一届政府面临着新形势、新任务的考验，也承担着组织和人民的重托。因此，我们必须全面加强自身建设，充分体现和展示新作风、新作为、新形象。

　　一是建设发展型政府，坚持科学发展，做到不动摇、不折腾。要进一步统一思想认识，坚持既定发展战略，切实做到以经济建设为中心，以加快发展为主要矛盾，以产业项目为主要抓手，不因任何变化、任何困难而改变方向和目标。二是建设责任型政府，坚持知责尽责，做到不推诿、不退缩。要把高度负责、敢于担当作为政府每个成员的核心素质和基本要求，无论是日常工作还是急难险重任务，都能展示良好的胜任力和执行力，特别是对群众的难题、群众的疾苦，必须及时到位解决。要认真执行民主集中制，自觉接受人大、政协和人民的监督，健全决策评估、目标管理等制度，对失职渎职者要坚决追责问责。三是建设实干型政府，坚持真抓快干，做到不懈怠、不浮夸。要树立正确的政绩观和价值观，把实干作为政府的集体风格，坚持人人、事事讲效率、抓落实、求实效。做到干部工作在一线、政策落实在一线、问题解决在一线、成效检验在一线，班子成员每年深入实际现场办公和调查研究的时间不少于两个月。四是建设服务型政府，坚持执政为民，做到不忘本、不作秀。要把维护和保障人民群众利益作为政府一切工作的出发点和落脚点，下大力气解决群众不满意的问题，真正把民生的承诺和口号落实到群众的衣食住行、柴米油盐上，让人民群众实实在在地享受到发展的成果。要坚持民生工作向市民征求意见制度，开展民生工作群众满意度测评活动。五是建设学习型政府，坚持提高能力，做到不平庸、不自满。要全员全面加强学习，提高本领，着力解决部分领导干部知识、能力、观念不适应等问题，提倡政府班子成员日均至少学习一小时。要大力推动改革创新，敢争敢闯，不断提升政府各项工作在全省的位置，对长期打不开局面的干部进行群众评议，并根据评议结果进行岗位或分工调整。六是建设廉洁型政府，坚持严守法纪，做到不逾矩、不腐败。要切实加强换届后班子思想政治建设，突出解决一些领导干部在是与非、公与私、真与假、实与虚等方面存在的问题。坚持“一岗双责”和“三重一大”制度，严禁乱伸手、乱插手、乱说话。要全员提升官德修养，做到以德施政、以廉服众，树立良好的政府形象。以上六条，政府全体同志要互励共勉，也恳请人大、政协和全市人民监督检验。

　　各位代表，发展的蓝图已绘就，奋斗的目标已明确。在未来的五年里，我们一定以人民幸福为第一责任，以人民满意为第一标准，励精图治、奋发图强、不负众望、不辱使命，努力把我们这座传统煤城建设成更有实力、更有活力、更有魅力的新鹤岗！
